# Barriers, enablers, and opportunities for organisational follow-up of workplace violence from the perspective of emergency department nurses: a qualitative study

**DOI:** 10.1186/s12873-021-00413-7

**Published:** 2021-02-12

**Authors:** Brodie Thomas, Anthony McGillion, Kristina Edvardsson, Peter O’Meara, Julia Van Vuuren, Evelien Spelten

**Affiliations:** 1 La Trobe Rural Health School, La Trobe University, 471 Benetook Ave, Mildura, VIC 3500 Australia; 2 School of Nursing and Midwifery, La Trobe University, Plenty Rd & Kingsbury Dr, Bundoora, VIC 3086 Australia; 3 Judith Lumley Centre, La Trobe University, Melbourne, Australia; 4 Department of Emergency Health and Paramedic Practice, Monash University, McMahons Road, Frankston, VIC 3199 Australia

**Keywords:** Emergency service, hospital, Nurse administrators, Nurse managers, Workplace violence, Risk management, Organizational objectives

## Abstract

**Background:**

A lack of follow-up of violence incidents and assaulted staff has been associated with high levels of workplace violence. There is a paucity of literature on the barriers, enablers and opportunities for organisational follow-up of workplace violence. The aim of this study was to explore the barriers, enablers and opportunities for organisational follow-up of workplace violence from the perspective of Emergency Department nurses.

**Methods:**

This qualitative study comprised two focus groups with Emergency Department nurses. Data were analysed thematically. COREQ guidelines were followed for the design and reporting of the study.

**Results:**

The barriers to follow-up in this study relate to the type of perpetrator, the initial incident response, the incident reporting process and organisational action. The enablers included hospital initiatives to manage violence and support staff wellbeing. The opportunities included strategies to improve follow-up and ideas for new follow-up strategies.

**Conclusions:**

Organisational follow-up is important for the emotional and professional wellbeing of staff who experience workplace violence. Opportunities for follow-up include exploring different approaches to patients with mental health issues and focussing on reoffenders by providing appropriate support and consequences. Managers should advocate for efficient and standardised reporting processes and ensure assaulted staff have a clear perception of follow-up and are included in the follow-up process. Including the perpetrators in the follow-up process may reduce workplace violence.

**Supplementary Information:**

The online version contains supplementary material available at 10.1186/s12873-021-00413-7.

## Background

Workplace violence directed at healthcare workers from patients and bystanders is a persistent worldwide problem [1]. This phenomenon appears to be increasing in frequency and severity, and has significant implications for all levels of the healthcare system [2]. Workplace violence has a negative impact on individual worker’s physical and psychological health, social and financial well-being, and job satisfaction [3–5]. Healthcare organisations and communities are also impacted by violence in healthcare, through the economic impact, staff turnover, and a decrease in professional competence and quality of care [3–5].

The Emergency Department (ED) environment poses challenges to the prevention and management of workplace violence. Compared to other hospital departments, the ED patient population is heterogeneous, staff are less likely to have a familiar relationship with patients, and those visiting the department are often in the midst of a crisis or dealing with high levels of stress [6]. In Australia, there has been an increase in ED presentations without any proportionate increase in staff levels, which leads to overcrowding [7]. ED overcrowding is a global healthcare issue that increases the frequency of workplace violence [8].

Many interventions have been developed to address workplace violence in healthcare. The majority focus on the education and training of staff, particularly de-escalation strategies, yet there is very limited evidence to support the effectiveness of any interventions [1]. An essential part of effective violence prevention programs that is not a focus of scholarly literature is organisational follow-up. In this study, the concept of follow-up refers to the post-incident response and evaluation and is defined as consisting of four main components: 1) Assistance and support for assaulted staff; this includes medical treatment, psychological and social support. This support should be provided both as an immediate response and ongoing to address the long-term consequences of violence [9–11]. 2) Incident reporting; recording an incident is crucial for the follow-up process to work. Reporting procedures are recommended to be easy, not time consuming and encouraged by management [11, 12]. 3) Evaluation of the incident; investigation of the violent incident should occur with the principles of risk mitigation and potential review of current interventions. 4) Information and communication; keeping staff members informed regarding the incident and the follow-up process [11]. Follow-up not only aims to improve reporting but to reduce the negative consequences of violence on both the victims and the workplace [11]. There is a paucity of literature on the organisational follow-up of workplace violence aside from stating its value [10, 13–18]. A lack of follow-up has been associated with high levels of workplace violence and an absence of accountability for perpetrators [17, 19, 20]. Studies of organisational follow-up in ED’s have been recommended for investigation [21].

### Study aim

This study aimed to investigate ED nurses’ perspectives and experiences of the barriers, enablers and opportunities for organisational follow-up of violent incidents in the ED.

## Methods

### Study design

A constructivist research paradigm guided this investigation into the subjective and socially constructed reality of the participants [22]. A qualitative approach was used with focus groups as the data collection method to explore the meaning and the significance of the participant’s experiences [23].

### Study setting

The study took place at a large metropolitan hospital in Victoria, Australia. The selected hospital has identified the reduction of workplace violence as a priority and implemented several interventions which are described in Table 1 [24].

**Table 1:** Interventions to address workplace violence implemented by the studied hospital [24–28]

Program	Description
RiskMan	A reporting system to record, notify and investigate health and safety incidents and near misses at work.
Tap Out program	Nurses nominate to be swapped from one area of the ED to another in order to remove them from challenging patients or situations.
Muster Slides	Highlight potentially challenging patients which allows the in-charge nurse to distribute them evenly among nurses to reduce compassion fatigue and mental injury.
Accountability letter	A letter is sent on behalf of the hospital to a perpetrator of violence, informing them that their behaviour was inappropriate and that an alert will be placed on their medical record.
Behavioural assessment unit	The unit is co-located with the ED but removes patients from the more chaotic environment of the main ED area. The aim is to fast-track the admission of patients with intoxication and mental health issues.
Management plan	Management plans for complex patients include tailored strategies to prevent aggressive behaviour and de-escalate the specific patient when they become aggressive.

### Participant recruitment

Recruitment took place in November 2018. The participants, all ED nurses, were taking part in an education day at the hospital as part of their continuing professional development. The participants were informed prior to the day that the focus groups on this subject were being conducted. On the day, the nurse manager invited staff to participate and it was not known who declined to participate. The interview guide was explained to the participants prior to the focus groups being conducted. Eighteen nurses participated in the study. None of the nurses declined participation or dropped out of the study.

### Data collection

The focus groups were conducted in November 2018 at the hospital. Only the research team and participants were present during the focus groups. A semi-structured interview guide was developed by the research team to facilitate discussion of incident reporting, organisational follow-up and perpetrators of violence. The guide is summarized in Table 2. Two focus groups were moderated by one member of the research team while three other members took field notes and ensured all participants were included in the discussions. Two focus groups with 10 and 8 participants were conducted as the hospital facilitated two time slots. Each focus group was allocated 90 min in which all of the discussion topics were discussed. The focus groups were audio recorded. The topic of this paper is the identified themes concerning organisational follow-up.

**Table 2:** Focus Group Interview Guide

Focus Group Interview Guide	
1. Experience of reporting violence at work	
2. The new reporting system	
3. Perception and approach to perpetrators	
4. Working with perpetrators to prevent violence	
4.1. Accountability letter	

### Data analysis

Audio recordings were transcribed verbatim. The transcripts were read and coded by four members of the research team using NVivo software [29]. The data were coded through a process of open, axial, and selective coding. Inductive thematic analysis was used to derive the themes from the data [30, 31]. Themes related to organisational follow-up were sorted into three categories: barriers, enablers and opportunities. The theoretical model inspired by Grol and Wensing [32] was used to organise the results of the study and was not used to guide the inception or analysis of the study. This is presented in Table 3. Grol and Wensing’s [32] theoretical model was created by combining 13 theories for change implementation in the healthcare system. The model identifies six different levels of the healthcare system to consider: the innovation (interventions); professionals; patients (perpetrators); social context; organisational context; and economic and political context [32, 33]. This model has been used to evaluate interventions for workplace violence in healthcare [12].

**Table 3:** Barriers, enablers and opportunities of follow up for workplace violence

Level	Barriers	Enablers	Opportunities
**Individual** Professionals	Incident reporting process: • Lack of reporting • Experience with police	• Staff injuries	
**Individual** Perpetrators	Type of perpetrator: • Complex patients, reoffenders and visitors		• Different approach: Prevent mental health patients in ED
**Social Context**	• High frequency violence		• Public education
**Organisational Context**	Initial response: • Accessing previous risk behaviour • Rewarding bad behaviour • Technical problems to report events Organisational action: • Lack of active follow-up	• Organisational culture and support • Tap Out program • Muster Slides • Management plan • Recording risk behaviour • Accountability letter	• Consequences for perpetrators • Apology letter • Security footage • Easier reporting process • Feedback to staff
**Economic and Political context**			
**Innovation (interventions)**			

### Reporting

To improve the quality and transparency of reporting the methods and results, the consolidated criteria for reporting qualitative research (COREQ) were followed [34]. The completed COREQ checklist can be found in the supporting information (Additional file 1).

### Ethics approval and consent to participate

Ethical approval was obtained from the Melbourne Health Human Research Ethics Committee under number QA2018132. Participants were provided with a participant information statement and informed consent was obtained from all participants prior to conducting the focus group. Due to the considerable number of participants in each focus group, informed consent was verbal, and audio recorded. The ethics committee considered verbal consent appropriate and approved this procedure.

### Research team and reflexivity

Three members of the research team have a PhD, two are PhD candidates and one is a research assistant. Three members of the team are male and three are female. All members of the research team have experience conducting qualitative studies. The participants had no knowledge or relationship with the researchers prior to the focus groups. The research team are involved in several studies of workplace violence in healthcare.

## Results

### Participant characteristics

The age of participants ranged from 18 to 59 years, with the majority (*n* = 8) ranging between 30 and 39 years. Their experience ranged from less than 1 year to over 15 years, with the majority (*n* = 11) having less than 10 years’ experience in the ED. Sixteen of the 18 participants were female (*n* = 16), which is representative of the nursing workforce in Australia [35].

### Barriers, enablers, and opportunities for organisational follow-up

Eight barriers, seven enablers, and seven opportunities for organisational follow-up were discussed. Table 3 provides an overview of these themes applied to a model inspired by Grol and Wensing [32]. No enablers were highlighted at the perpetrator or the social level and no opportunities were highlighted at the professional level. No themes were identified at the Economic and political context, and Innovation of interventions levels.

#### Barriers to follow-up

A key barrier at the *individual professional level* was a high tolerance of workplace violence among staff. The high frequency of violent events resulted in staff not reporting all incidents. Without incident reporting, there is no follow-up of violence incidents.

*“We have a very high threshold, we tolerate so much, that it has to be a significant incident or a significant threat before we actually do something … it’s just water off a duck’s back, to be abused, threatened … ”* (Participant 10)

Another barrier was the participants’ experience with Police, as apathy from Police officers had prevented some participants from reporting incidents. They recounted stories of being told not to bother reporting because no action would be taken. A lack of feedback from Police discouraged future incident reporting, as staff members never found out the outcome of previous reports.

*“This system itself let me down. I wasn’t supported. And I went to the police and they basically turned a blind eye with some other associated issues that were out of their hands.”* (Participant 4)

In contrast, two participants found Police to be supportive and indicated that they would be more likely to report again. They highlighted that education sessions from Police convinced them that they should report more.

*“The police officer saying something during the week, I was like, actually we do need to report this. That played a role.”* (Participant 6)

The most prevalent type of perpetrators acting as barriers at the *individual perpetrators level* were described as complex patients, often with challenging mental health and behavioural issues, including reoffenders.

*“They’re frequent flyers. They come in and unfortunately they do have these personality disorders or anti-social personality disorders where they … they’re not deterred, and they thrive on conflict.”* (Participant 8)

The participants did not believe that these perpetrators responded well to the accountability letter initiative described in Table 1 and might even provoke further violent behaviour.

*“They sent this man a letter saying, ‘we’re acknowledging what has happened and … we have a zero tolerance’. This man has an anti-social personality disorder, I almost feel like this is going to fuel him more because he was so angry that we hadn’t helped him.”* (participant 8)

Visitors to the ED, including patient advocates, were also identified as barriers as the staff members did not often have a rapport with them and did not know how to report them.

*“It’s hard with family though, because I find it difficult to report. I want to report an incident, and I find it hard to do because they are not actually registered on the system, they don’t have any details about them, who they were.”* (Participant 12)

The barrier at the *social context level* was the high frequency of violent events. The participants all agreed that the current incident reporting system was too inefficient to report and investigate the copious number of incidents.

*“We have multiple [security alerts] a shift and attached to one of those is meant to be a Riskman … I could confidently say that when a code grey is called, no stat … if you call 15 code greys a day, there is no way you get 15 Riskmans associated with that. You might be lucky to get one.”* (Participant 4)

Four barriers were highlighted at the *organisational context level*.

##### Accessing previous risk behaviour

Some perpetrators have an alert placed on their medical records to inform staff of their history of violence behaviour. The triage nurse can access this information, however the participants pointed out that this information is rarely available before nurses assess them.

*“Flagging these patients is a big thing for us, so we can identify as soon as these people rock up to triage, that we can say, he’s a high risk … but you know, sometimes you don’t know until it’s too late”.* (Participant 8)

##### Rewarding bad behaviour

Several participants explained that many perpetrators appear to use violence deliberately in the ED to achieve specific goals such as skipping long waits in the waiting room. They expressed their frustration that often such behaviour is rewarded by staff facilitating the requests. This was highlighted as a barrier as the situations are resolved and do not get investigated, however the perpetrators learn that violence will be tolerated and can be a useful tool in the ED.

*“They scream and rant in the waiting room, and suddenly, bang (snaps fingers), they’re in the door, they get seen, they get sorted. Because nobody wants a carry-on.”* (participant 17)

##### Technical problems to report events

The participants described the incident reporting system RiskMan as a barrier. The fundamental issues raised with the RiskMan system were that it is was too difficult and time consuming to complete.

*“The Riskman is too difficult, we don’t bother … You write in the notes ‘patient aggressive, code grey called’, and then you talk about the outcome with the code grey. But when it comes to Riskman, I don’t think we have any studies at all, or any evidence at all to show that we have code grey here in the department.” (Participant 3)*

##### Lack of active follow-up

Participants argued that there is often a lack of organisational action and feedback following violent incidents. This exacerbated the issue of follow-up as the participants did not see the value in reporting when it appeared that no action would be taken based on their reports.

*“The other thing is that what is reported doesn’t ever get any feedback … We don’t know what the repercussions were to the person that was abusive.”* (Participant 7)

#### Enablers to follow-up

The only enabler at the *individual professional level* were incidents that result in injuries to staff. The participants said that they always report incidents involving injuries as it may be required if they take time off work.

*“This is probably a bad attitude … but you have to have a serious injury or something like that to then do a RiskMan.* (Participant 8)

*“For potential work cover”* (Participant 3)

The participants described six enablers at the *organisational level*.

##### Organisational culture and support

Many of the participants felt that the culture of the hospital had changed to a stronger focus on staff wellbeing. Management encouraged them to report all incidents of workplace violence and the support for staff that were injured had improved.

*“Culture-wise they’ve really put a lot of effort in trying to say you will be supported. Our boss wants to be told, she’s not someone that would discourage you telling her”.* (Participant 8)

##### Muster slides and tap out program

As described in Table 1, the purpose of both initiatives was to prevent staff becoming overwhelmed and emotionally fatigued. Many participants felt supported knowing they had the option to be moved away from challenging patients, while others felt they would be unlikely to utilise the Tap Out process as another staff member would take their place.

*“Yeah, I’d like to think I’d take advantage of it but like everyone else said, you don’t want to be putting someone else in that position. But it is nice to know in the back of your mind that if you’re really just can’t handle it anymore, there is the option to swap.”* (Participant 7)

##### Management plans

The participants found management plans to be valuable when managing difficult patients and felt supported as they could see that action was being taken following violent incidents. The downside of management plans is that they are very slow to implement and take multiple presentations.

*“It’s a very long process to get the management plan. So once that management plan is done … to me it’s a relief.”* (Participant 3)

##### Recording risk behaviour

The general consensus was that recording the violent behaviour of perpetrators and having this available for future presentations is valuable and helps staff to prepare for potentially violent patients. The participants referred to this process as “flagging” as a red flag is placed on the patient record.

*“Flagging these patients is a big thing for us, so we can identify as soon as these people rock up to triage, that we can say, he’s a high risk … but you know, sometimes you don’t know until it’s too late”.* (Participant 8).

##### Accountability letter

The participants believed the accountability letter would be useful for perpetrators that were ‘not their normal selves’ during the incident; for example, being impacted by stress, a mental health crisis or more commonly, drugs and alcohol. These perpetrators were described as being able to reflect on their actions, apologetic, and upset by their behaviour. The participants did not believe this initiative would work for complex patients with challenging mental health or behavioural issues.

*“It depends on who it is … Unfortunately, it’s probably only beneficial to some people. But I think everybody needs to be accountable and at least this is something that you can say ‘you’ve been told’ so you can’t pretend … ”* (Participant 8)

#### Opportunities for follow-up

The discussion on opportunities at the *individual perpetrator level* revolved around limiting the time perpetrators spent in the ED. Mental health patients can spend very long periods of time in the ED waiting for a mental health assessment or for a bed in a psychiatric ward. While this is not directly related to follow-up, the participants felt it has a considerable influence on violence in the ED and an answer may be found in the follow-up of these patients. They highlighted that these patients need to be managed and followed up differently to other patients, contrary to current practice.

*“I completely agree with that patient, it’s the frustration. You’re simply going ‘I know’ and it’s EMH (mental health team) that’s delayed’ or … ‘you can’t leave because you’ve been under a section 351’. You can say these … until EMH arrive, you can only tell her so many times. You can’t hurry them up.”* (Participant 3)

*“She had been waiting 16 hours as well, so she had been here for some time before she started to escalate.”* (Participant 6)

*“The (mental health clinician) knew her … and de-escalated it straightaway. The behaviour then stopped. It’s the knowledge about people, their access and appropriate mental health or care plan.”* (Participant 15)

Public education was highlighted as an opportunity at the *social context level*. Participants explained that there is considerable confusion among visitors about how ED’s work and which behaviours are appropriate.

*“There is a lack of education in the community about how EDs actually work. You know, it’s not a first in, first dressed. There are different streams, there are waiting times for those streams … they need to know what emergency is and what’s prioritised. It’s just a lack of education in the community.”* (Participant 9)

Follow-up from previous violent episodes had led to a violence prevention video being created and played in the waiting room at this hospital. Participants felt it was an effective form of follow-up and believed it had an impact in reducing violence.

*“I found it very beneficial because people would be [getting aggressive] and they would then watch the video … ”* (Participant 12)

Five opportunities for follow-up were highlighted at the *organisational level*.

##### Consequences for violent behaviour

The participants noted that some perpetrators appear to be rewarded for violent behaviour in the ED when there are no consequences for their behaviour. The participants believed that having consequences for their negative actions may break a cycle of violent behaviour, and at the very least, not reward violent behaviour.

*“what happens … a lot, is you say somebody is being verbally abusive, it happens a lot at triage, and a lot from relatives. You escalate it, but what happens is there is no comeback on them apart from their issue is resolved a lot quicker. Instead of it just being a clear cut ‘no, this is not how you behave … ’. What they want happens. And I find that … that’s just actually encouraging (violent) behaviour, even though we say we don’t have the tolerance, there is a massive tolerance already.”* (Participant 15)

##### Apology letter from the perpetrators of violence

Several participants felt that an apology from perpetrators would mean a lot to them, especially for the perpetual everyday abuse. They did stress that in general, they would not like to meet the perpetrator again and would prefer to receive a letter with a sincere apology.

*“Yeah, I don’t know … in all honesty, I wouldn’t really want to see this guy again.”* (Participant 8)

*“They could write a letter themselves, you know. Like document it.”* (Participant 9)

##### Use security video footage during the follow-up process

The participants believed that security footage would be a valuable tool for the follow-up process.

*“I have a really great rapport with (security), so I can pull up the video and I can have a look at it. But that is actively discouraged by the hospital. If they get caught, they get told off … But there is a resource there that we’re not allowed to use.”* (Participant 3)

*“I think they use them … when it was popular in the news, that was all footage … a lot of that was from us... If an incident happened, it would always be there to use.”* (Participant 8)

##### Quick and easy process

All participants agreed that the reporting of workplace violence in the ED would be enhanced by a more streamlined process. A document similar in nature to the patient history would be ideal; a reporting process which can be written freely, without the need to select excessive drop boxes, and could be completed in real time alongside the patient notes. In addition, a document specifically addressing violence in the ED would be more effective than the complicated RiskMan process.

*“Quick and easy … something you can do then and there, present time. Maybe say, 5 minutes, I’ve got to sit down and do this.”* (Participant 11)

##### Providing feedback to staff

The participants said they would like to see action taken as a result of their reports and be informed of the outcome. This would encourage more consistent reporting of incidents.

*“The follow-up is unclear, which doesn’t encourage you to do anything about it as well … Feedback would encourage reporting, if you knew some follow-up, through the police or your own system” (*Participant 17)

## Discussion

### Main findings

In this study we explored the barriers, enablers and opportunities for follow-up of workplace violence from the perspective of ED nurses. The focus groups revealed eight barriers, seven enablers and seven opportunities for follow-up. Using the Grol and Wensing model [32] to structure the results, it became clear that barriers were highlighted at multiple levels of the healthcare system while almost all of the enablers and opportunities were at the organisational level. The barriers to follow up related to the type of perpetrator, the initial response to the incident, the incident reporting process and organisational action. The identified enablers largely related to the initiatives that the hospital had put in place to manage violence and support staff wellbeing. The opportunities included both strategies to improve follow-up as well as ideas for the way follow-up might occur.

### Interpretation of findings

Organisational follow-up should be a priority for nursing managers, completed consistently, and include the affected staff. It has been shown to have a number of important effects on the negative impact of workplace violence. The strongest influences appear to be on emotional wellbeing of assaulted staff [10, 14, 16]. The focus groups demonstrated that when follow-up occurs, nurses feel supported by their managers and may be more resilient against emotional fatigue. Follow-up has been previously shown to improve job satisfaction, prevent burnout and turnover intention, and support professional competence and quality of care from staff who have experienced violence [14–16]. The focus on follow-up with assaulted staff should not only be on their immediate response to violence but should also include long-term monitoring and support [9]. The participants in this study found that follow-up leading to the creation of behavioural care plans was valuable as they facilitate de-escalating patients who are becoming violent and aggressive.

Almost all of the enablers for follow-up were organisational initiatives that show action and support from management. However, despite the significant value of follow-up described in the focus groups and in the wider literature, the experience of many participants in this study indicate they were not included in the follow-up process. It would be valuable for the staff members to be aware of the evaluation and learning that occurred following violent incidents. Organisational follow-up also appears to improve staff motivation to report violent incidents [12, 17, 36]. Healthcare organisations and managers must be mindful that this requires an efficient incident reporting system. A standardised incident reporting system that is simple, time efficient and can enable sharing of data across organisations would be ideal [12, 17, 18].

During the focus groups participants expressed the view that future violence may be reduced by including the perpetrators of violence in organisational follow-up. The two most common cohorts discussed were mental health patients and reoffenders.

The ED is a counterproductive environment for patients with mental health issues. EDs are often chaotic, overstimulating, and have excessive waiting times for involuntary patients waiting for psychiatric evaluation. These patients may not have their psychiatric conditions managed optimally while waiting for admission to a psychiatric facility, instead receiving medications and restraint to manage their aggression. Despite these issues, studies from Canada and the United States report that patients continue to attend general EDs for mental health concerns as they feel they have no alternative [37–40]. In order to address this issue, there is a growing area of research proposing alternative models of mental health service delivery. While the research into community-based models of care for mental health emergencies has shown promising results and the follow-up of patients with these services may prevent them presenting to the ED, the long-term effectiveness remains unclear [40–42].

Reoffenders are overrepresented in statistics of violence in EDs and they dominated much of the focus group discussions. Focus group participants described the impunity the majority of violent reoffenders receive and expressed their desire for appropriate consequences for violent reoffenders. This is similar to other professionals working in emergency healthcare [12]. While there is little evidence regarding consequences for violent offenders in healthcare literature [1], criminology studies provide some evidence that certainty of sanctions are a greater deterrent to reoffenders than increasing the severity of punishment [43]. This notion is similar to what the participants described; they want consequences to be consistently applied to perpetrators of violence. Criminology has a strong focus on reoffenders, often termed recidivists [44]. The description of recidivists in criminology research is strikingly similar to the reoffenders of violence discussed in healthcare [44, 45]. While this cohort is notoriously difficult to manage, criminology studies indicate that criminal risk can be reduced in reoffenders. The majority of research in this field focuses on cognitive behavioural therapy programs [44]. The evidence base for reducing recidivism in criminology is similar for interventions to reduce violence in healthcare, with a lack of reliable evidence to form a consensus [46].

### Strengths and limitations

The large size of the focus groups was a potential limitation as some participants engaged in discussions more than others. Participants’ exclusion from conversations was minimised by the presence of four researchers observing participant engagement. Another limitation was the participants were all nurses from one hospital, which may limit the transferability of results. Gaining broader perspectives on this topic from other staff such as doctors and security personnel as well as other data sources such as the documentation of incident data and subsequent follow-up might help gain a better understanding of organisational follow-up.

## Conclusion

Organisational follow-up is important for the wellbeing of staff and for improving the reporting of workplace violence. Opportunities for follow-up include investigating different approaches to patients with mental health issues such as engaging them in appropriate community-based services and focussing on reoffenders by providing appropriate consequences and support. For the greatest benefit from follow-up, staff should have a clear perception of follow-up and support and be included in the process.

## Supplementary Information

**Additional file 1.**

## Data Availability

Focus group data are available via Figshare (10.26181/5e2f5d7dbf3e8).

## Abbreviation

ED: Emergency department
